# *Leptospira* infection in rats: A literature review of global prevalence and distribution

**DOI:** 10.1371/journal.pntd.0007499

**Published:** 2019-08-09

**Authors:** Kenneth Boey, Kanae Shiokawa, Sreekumari Rajeev

**Affiliations:** Ross University School of Veterinary Medicine, Basseterre, St. Kitts, West Indies; Mahidol University, THAILAND

## Abstract

**Background:**

The role of rodents in *Leptospira* epidemiology and transmission is well known worldwide. Rats are known to carry different pathogenic serovars of *Leptospir*a spp. capable of causing disease in humans and animals. Wild rats (*Rattus* spp.), especially the Norway/brown rat (*Rattus norvegicus*) and the black rat (*R*. *rattus*), are the most important sources of *Leptospira* infection, as they are abundant in urban and peridomestic environments. In this study, we compiled and summarized available data in the literature on global prevalence of *Leptospira* exposure and infection in rats, as well as compared the global distribution of *Leptospira* spp. in rats with respect to prevalence, geographic location, method of detection, diversity of serogroups/serovars, and species of rat.

**Methods:**

We conducted a thorough literature search using PubMed without restrictions on publication date as well as Google Scholar to manually search for other relevant articles. Abstracts were included if they described data pertaining to *Leptospira* spp. in rats (*Rattus* spp.) from any geographic region around the world, including reviews. The data extracted from the articles selected included the author(s), year of publication, geographic location, method(s) of detection used, species of rat(s), sample size, prevalence of *Leptospira* spp. (overall and within each rat species), and information on species, serogroups, and/or serovars of *Leptospira* spp. detected.

**Findings:**

A thorough search on PubMed retrieved 303 titles. After screening the articles for duplicates and inclusion/exclusion criteria, as well as manual inclusion of relevant articles, 145 articles were included in this review. *Leptospira* prevalence in rats varied considerably based on geographic location, with some reporting zero prevalence in countries such as Madagascar, Tanzania, and the Faroe Islands, and others reporting as high as >80% prevalence in studies done in Brazil, India, and the Philippines. The top five countries that were reported based on number of articles include India (*n* = 13), Malaysia (*n* = 9), Brazil (*n* = 8), Thailand (*n* = 7), and France (*n* = 6). Methods of detecting or isolating *Leptospira* spp. also varied among studies. Studies among different *Rattus* species reported a higher *Leptospira* prevalence in *R*. *norvegicus*. The serovar Icterohaemorrhagiae was the most prevalent serovar reported in *Rattus* spp. worldwide. Additionally, this literature review provided evidence for *Leptospira* infection in laboratory rodent colonies within controlled environments, implicating the zoonotic potential to laboratory animal caretakers.

**Conclusions:**

Reports on global distribution of *Leptospira* infection in rats varies widely, with considerably high prevalence reported in many countries. This literature review emphasizes the need for enhanced surveillance programs using standardized methods for assessing *Leptospira* exposure or infection in rats. This review also demonstrated several weaknesses to the current methods of reporting the prevalence of *Leptospira* spp. in rats worldwide. As such, this necessitates a call for standardized protocols for the testing and reporting of such studies, especially pertaining to the diagnostic methods used. A deeper understanding of the ecology and epidemiology of *Leptospira* spp. in rats in urban environments is warranted. It is also pertinent for rat control programs to be proposed in conjunction with increased efforts for public awareness and education regarding leptospirosis transmission and prevention.

## Author summary

The role of rodents in the transmission of many diseases, including leptospirosis, is widely known. Rats abundant in urban and peridomestic environments are the most important reservoirs and sources of *Leptospira* infection in humans and animals. Leptospirosis is a significant but neglected disease of humans and animals that is increasing in incidence in regions affected by natural disasters. This paper summarizes the global prevalence and distribution of *Leptospira* infection in rats and will add to the literature that supports research, education, and public awareness regarding leptospirosis transmission and prevention.

## Introduction

Leptospirosis is a major zoonotic disease worldwide, having significant impact on both human and animal health [1, 2]. It is known to be the most widespread zoonosis in the world [3], affecting an estimated 1.03 million people and causing 58,900 deaths annually [4]. Leptospirosis can also cause major economic losses in livestock industries because of abortions and stillbirths in farm animals [2]. The disease is caused by pathogenic spirochete bacteria of the genus *Leptospira*, which consists of 22 known species (pathogenic, intermediate, and saprophytic) and is divided into more than 300 serovars [5]. Recently, 12 novel species of *Leptospira* have been isolated from tropical soils, suggesting a highly unexplored biodiversity in the genus [6].

Not only is leptospirosis a public health issue in developing countries, it has become an urban health problem in developed and industrialized countries, occurring in unsanitary environments in cities during periods of seasonal rainfall and flooding [7]. Leptospirosis is also associated with natural disasters, with large outbreaks occurring after hurricanes, typhoons, and floods in tropical regions [8].

A wide variety of mammals can act as reservoirs of *Leptospira*, harboring pathogenic *Leptospira* spp. in their renal tubules and then shedding them through urine, thus contaminating the environment [1]. Humans and other animals may be exposed to *Leptospira* spp. by direct or indirect contact with infected animals or through the contaminated environment such as soil or water [1, 9]. Vertical transmission from mother to fetus or neonate through transplacental or transmammary transmission, respectively, as well as through sexual transmission within species, may also occur [2].

Wild rats (*Rattus* spp.), especially the Norway/brown rat (*Rattus norvegicus*) and the black rat (*R*. *rattus*), are abundant in urban and peridomestic environments and are the most important known sources of *Leptospira* infection [1, 10]. Rats are chronic asymptomatic carriers of *Leptospira* spp., maintaining the spirochetes in their proximal renal tubules [11, 12]. They have also been reported to carry different pathogenic serovars of *Leptospir*a spp. capable of causing disease in humans and other animals [13].

In this study, we focused on compiling and reviewing available data in the literature on global prevalence of *Leptospira* exposure and infection in rats, as well as comparing the global distribution of *Leptospira* spp. in rats with respect to prevalence, geographic location, methods of detection, diversity of serogroups/serovars, and species of rats.

## Methods

### Literature search

To find studies describing *Leptospira* prevalence in rats worldwide, a thorough literature search was conducted using PubMed (https://www.ncbi.nlm.nih.gov/pubmed) with search terms including but not limited to “((*Leptospira*) OR (Leptospirosis)) AND (Rats) AND (Prevalence),” “((*Leptospira*) OR (Leptospirosis)) AND (*Rattus*) AND (Prevalence),” “((*Leptospira*) OR (Leptospirosis)) AND (Rats) AND (Seroprevalence),” “((*Leptospira*) OR (Leptospirosis)) AND (*Rattus*) AND (Seroprevalence),” “((*Leptospira*) OR (Leptospirosis)) AND (Rodents) AND (Prevalence),” and “((*Leptospira*) OR (Leptospirosis)) AND (Rodents) AND (Seroprevalence),” without restrictions on publication date. In addition, a search was performed using internet-based search engines such as Google and Google Scholar using similar search terms to manually search for other relevant articles.

### Inclusion and exclusion criteria

Titles and abstracts were initially screened against the inclusion criteria to determine their suitability to be included in this review. Abstracts were included if they described data pertaining to *Leptospira* spp. in rats (*Rattus* spp.) from any geographic region around the world, including reviews. Abstracts were excluded if they did not describe the prevalence of *Leptospira* spp. in rats or if they did not describe naturally occurring *Leptospira* infection in the rats. The full text documents were then assessed against specific inclusion criteria. Publications in languages other than English were excluded; however, such articles with an English abstract were included if they contained relevant data for extraction.

### Data extraction

We extracted data including the authors, year of publication, geographic location, and methods of detection used from the articles retrieved. We also extracted information on the species of rat(s), sample size, and prevalence (any kind including sero/molecular/culture/other prevalence) of *Leptospira* spp. (overall and within each rat species) and information on species, serogroups, and/or serovars of *Leptospira* spp. detected if available. For cases in which mice or other rodents were also included in the study, we extracted only specific data regarding rats from the *Rattus* genus. We used a data extraction form to record the relevant data.

## Results

### Literature search

The database search retrieved 303 articles. Of these, 114 were rejected as duplicates, and 36 articles were excluded, as they did not have any information about *Leptospira* spp. in rats. Additionally, 29 articles that did not describe prevalence of *Leptospira* spp. in rats were excluded. Twelve publications in languages other than English were also excluded. However, six foreign language articles with an English abstract containing relevant data were included. Three articles describing prevalence of *Leptospira* spp. in laboratory rats, as well as four articles describing cases of human leptospirosis due to transmission from pet rats, were excluded for the purpose of this review; however, they will be discussed separately in this paper. In addition, 11 relevant articles were manually included through general internet-based searches, and a further 25 articles that reported *Leptospira* prevalence in different types of rodents or small mammals but had data on rats of the *Rattus* genus were included. In total, 145 articles published up until June 2018 were included in this literature review. Publication references are listed in S1 List.

Including articles in foreign languages (157 total), five articles were published before 1950, eight in 1951–1959, six in 1960–1969, five in 1970–1979, eleven in 1980–1989, seven in 1990–1999, 32 in 2000–2009, and 83 in 2010–2018. Half of the number of articles in foreign languages (9/18) were published earlier in time, between 1949 and 1970. The number of studies investigating *Leptospira* spp. in rats generally increased throughout the years and increased significantly in the last decade.

### Geographic distribution of *Leptospira* prevalence

**Fig 1:**
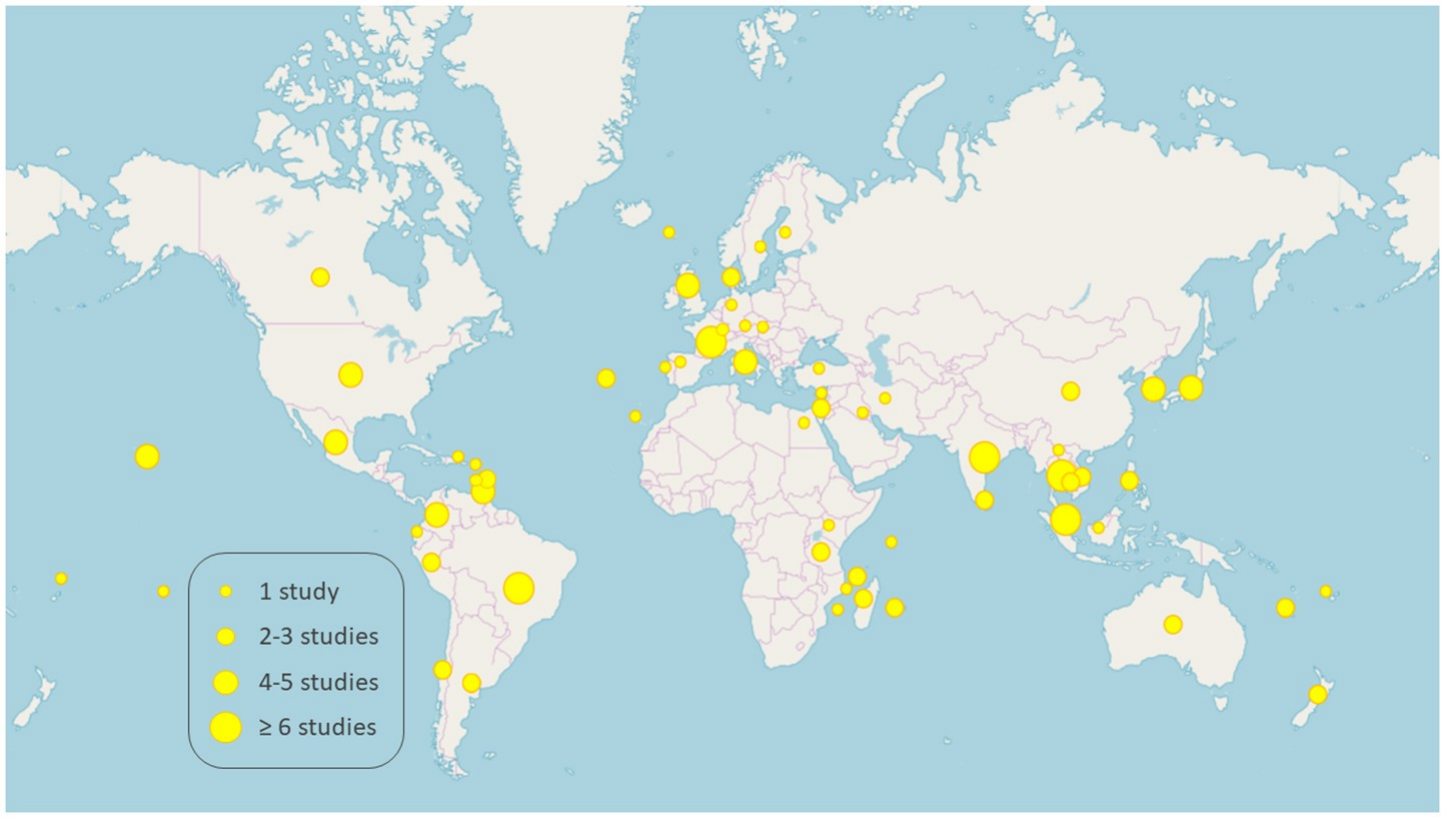
Geographic distribution of all 145 publications included in the literature review. Map template obtained from OpenStreetMap.

The publications retrieved reported relevant data from a total of 62 geographical locations (Fig 1; S1 Map). Data such as prevalence ranges and number of studies retrieved per geographic location are provided in Tables 1–8, grouped according to geographical continents or regions. Detailed data on prevalence and serogroup/serovar information from each publication can be found in S1 Table.

**Table 1:** Summary of *Leptospira* prevalence in rats in Oceania.

Geographic location	Number of articles	Seroprevalence	Molecular/culture/ other prevalence	Serovars detected	Rat species	References
Australia	3	0%–1.7%	0%–22.2%	Australis, Ballum	RN, RR, RF, RLu	[14–16]
Fiji	1	55.9%	ND	Australis, Bratislava, Autumnalis, Ballum, Bataviae, Copenhageni, Pomona, Pyrogenes	RN, RR, RE, RFr	[17]
French Polynesia (France)	1	ND	20.4%	ND	RN, RR, RE	[18]
Hawaii (United States of America)	4	24.1%–27.1%	16.0%–53.3%	Australis, Ballum, Icterohaemorrhagiae, Sejroë	RN, RR, RE	[19–22]
New Caledonia (France)	2	ND	20.1%–61.1%	Ballum, Canicola, Icterohaemorrhagiae	RN, RR, RE	[19, 23]
New Zealand	3	13.2%–27.6%	27.3%–31.9%	Ballum, Copenhageni, Pomona, Pyrogenes, Tarassovi	RN, RR	[13, 24, 25]
Wallis and Futuna (France)	1	ND	23.9%	ND	RN, RR, RE	[26]

Abbreviations: ND, no data; RE, *R*. *exulans*; RF, *R*. *fuscipes*; RFr, *R*. *frugivorus*; RLu, *R*. *lutreolus*; RN, *R*. *norvegicus*; RR, *R*. *rattus*

**Table 2:** Summary of *Leptospira* prevalence in rats in Asia.

Geographic location	Number of articles	Seroprevalence	Molecular/culture/ other prevalence	Serovars detected	Rat species	References
Cambodia	2	ND	9.6%–13.8%	ND	RN, RE, RAr, RT	[27, 28]
China	3	3.0%	0%–40%	Icterohaemorrhagiae	RN, RL, RT, RNi, RFl	[29–31]
India	13	0%–51.4%	0%–58.3%	Australis, Autumnalis, Bataviae, Canicola, Grippotyphosa, Icterohaemorrhagiae, Javanica, Pomona, Pyrogenes, Hardjo	RN, RR, RH, RRf	[32–44]
Indonesia	1	ND	6.1%–25.3%	ND	ND	[45]
Japan	4	ND	3.4%–73.7%	Autumnalis, Grippotyphosa, Hebdomadis, Copenhageni, Icterohaemorrhagiae, Javanica, Pomona, Pyrogenes, Patoc	RN, RR	[8, 46–48]
Malaysia	9	0%–17.9%	0%–15.9%	Australis, Autumnalis, Ballam, Bataviae, Canicola, Djasiman, Grippotyphosa, Hebdomadis, Icterohaemorrhagiae, Javanica, Pyrogenes, Hyos, Andamana	RN, RD, RE, RAr, RB, RM, RRj, RS, RTm, RW	[49–57]
Philippines	2	30.0%–92.5%	43.4%	Australis, Autumnalis, Losbanos, Canicola, Grippotyphosa, Ratnapura, Hebdomadis, Copenhageni, Icterohaemorrhagiae, Poi, Pomona, Manilae, Hardjo, Tarassovi, Patoc, Semaranga	ND	[58, 59]
South Korea	4	0%–7.7%	ND	Canicola	RN, RR	[60–63]
Sri Lanka	2	7.9%–10.0%	0%–10.5%	Icterohaemorrhagiae, Copenhageni, Javanica	RR	[64, 65]
Thailand	6	0%–6.4%	0%–37.1%	Australis, Bratislava, Autumnalis, Ballum, Bataviae, Hebdomadis, Copenhageni, Icterohaemorrhagiae, Javanica, Pomona, Pyrogenes, Sejroë, Wolffi, Tarassovi, Patoc	RN, RR, RE, RAr, RL	[66–71]
Thailand, Cambodia, Laos	1	ND	5.9%	ND	RN, RE, RAr, RL, RT, RA, RNi	[72]
Vietnam	2	17.0%–22.0%	4.3%	Australis, Autumnalis, Bataviae, Canicola, Cynopteri, Hurstbridge, Copenhageni, Icterohaemorrhagiae, Javanica, Louisiana, Panama, Pomona, Pyrogenes, Tarassovi, Patoc	RN, RE, RAr, RT	[73, 74]

Abbreviations: ND, no data; RA, *R*. *andamanensis*; RAr, *R*. *argentiventer*; RB, *R*. *bowersi*; RD, *R*. *diardii*; RE, *R*. *exulans*; RFl: *R*. *flavipectus*; RH, *R*. *hinton*; RL, *R*. *losea*; RM, *R*. *muelleri*; RN, *R*. *norvegicus*; RNi, *R*. *nitidus*; RR, *R*. *rattus*; RRf, *R*. *rufescens*; RRj, *R*. *rajah*; RS, *R*. *sabanus*; RT, *R*. *tanezumi*; RTm, *R*. *tiomanicus* (*R*. *jalorensis*); RW, *R*. *whiteheadi*

**Table 3:** Summary of *Leptospira* prevalence in rats in the Middle East.

Geographic location	Number of articles	Seroprevalence	Molecular/culture/ other prevalence	Serovars detected	Rat species	References
Iran	1	22.4%	3.3%–11.3%	Australis, Autumnalis, Ballum, Cynopteri, Grippotyphosa, Copenhageni, Icterohaemorrhagiae, Lai, Hardjo, Sejroë	RN, RR	[75]
Israel	2	4.7%–9.2%	3.2%–13.1%	Ballum, Bataviae, Grippotyphosa, Hebdomadis, Icterohaemorrhagiae, Szwajizak, Andamana, Semaranga	RN, RR, RAx	[76, 77]
Kuwait	1	ND	16.3%	Canicola	RN	[78]
Lebanon	1	ND	5.7%–11.4%	Icterohaemorrhagiae	RN, RAx	[79]
Turkey	1	8.5%	0%–27.1%	Bratislava, Autumnalis, Icterohaemorrhagiae, Hardjo	RN	[80]

Abbreviations: ND, no data; RAx, *R*. *alexandrinus*; RN, *R*. *norvegicus*; RR, *R*. *rattus*

**Table 4:** Summary of *Leptospira* prevalence in rats in Africa.

Geographic location	Number of articles	Seroprevalence	Molecular/culture/ other prevalence	Serovars detected	Rat species	References
Canary Islands (Spain)	1	ND	20.3%	Copenhageni	RR	[81]
Egypt	1	75.9%	6.9%–24.0%	Canicola, Celledoni, Grippotyphosa, Icterohaemorrhagiae, Pomona	ND	[82]
Europa Island (France)	1	ND	4.2%	ND	RR	[83]
Juan de Nova Island (France)	1	ND	3.6%	ND	RR	[83]
Kenya	1	ND	9.3%	ND	RN, RR	[84]
Madagascar	2	12.0%	0%–60.5%	Canicola, Kuwait,	RN, RR	[85, 86]
Mayotte (France)	2	0%	9.9%–15.9%	ND	RR	[19, 87]
Réunion (France)	3	79.5%	36.3%–68.0%	Canicola, Cynopteri, Icterohaemorrhagiae, Mini, Panama, Sejroë	RN, RR	[88–90]
Seychelles	1	ND	7.7%	ND	RN, RR	[91]
Tanzania	2	50.0%	0%	ND	RR	[92, 93]

Abbreviations: ND, no data; RN, *R*. *norvegicus*; RR, *R*. *rattus*

**Table 5:** Summary of *Leptospira* prevalence in rats in Europe.

Geographic location	Number of articles	Seroprevalence	Molecular/culture/ other prevalence	Serovars detected	Rat species	References
Austria	1	ND	0%	ND	RN	[94]
Azores (Portugal)	2	55.0%	20.9%–26.4%	Aborea, Ballum, Icterohaemorrhagiae, Sejroë	RN, RR	[95, 96]
Denmark	2	ND	20.0%–52.5%	Icterohaemorrhagiae, Pomona, Sejroë	RN	[94, 97]
Faroe Islands (Denmark)	1	ND	0%	ND	RN	[98]
Finland	1	60.0%	9.5%–61.0%	Icterohaemorrhagiae	RN	[99]
France	6	36.1%–100%	0%–66.7%	Cynopteri, Copenhageni, Icterohaemorrhagiae, Magnus, Sejroë	RN	[7, 100–104]
Germany	1	ND	15.1%–17.2%	ND	RN	[94]
Hungary	1	ND	0%	ND	RN	[94]
Italy	4	18.2%–69.6%	29.9%–45.5%	Ballum, Icterohaemorrhagiae	RN, RR	[105–108]
Portugal	1	ND	50.0%	ND	ND	[109]
Spain	1	ND	5.9%	Icterohaemorrhagiae	RN, RR	[110]
Sweden	1	16.7%	ND	Icterohaemorrhagiae, Istrica	RN	[111]
Switzerland	1	ND	10.3%	ND	RN	[94]
United Kingdom	4	1.2%–3.9%	0%–41.7%	Bratislava, Icterohaemorrhagiae, Ballum	RN	[112–115]

Abbreviations: ND, no data; RN, *R*. *norvegicus*; RR, *R*. *rattus*

**Table 6:** Summary of *Leptospira* prevalence in rats in North America.

Geographic location	Number of articles	Seroprevalence	Molecular/culture/ other prevalence	Serovars detected	Rat species	References
Canada	3	0%–17.9%	11.1%–12.0%	Icterohaemorrhagiae	RN	[116–118]
Mexico	4	6.2%–15.0%	12.3%–73.3%	Bratislava, Grippotyphosa, Icterohaemorrhagiae, Hardjo, Wolffi, Tarassovi	RN, RR	[119–122]
USA	4	44.1%–65.3%	12.0%–45.5%	Copenhageni, Icterohaemorrhagiae	RN	[123–126]

Abbreviations: ND, no data; RN, *R*. *norvegicus*; RR, *R*. *rattus*

**Table 7:** Summary of *Leptospira* prevalence in rats in Central America and the Caribbean.

Geographic location	Nuber of articles	Seroprevalence	Molecular/culture/ other prevalence	Serovars detected	Rat species	References
Barbados	2	32.6%	0.6%–22.0%	Autumnalis, Bim, Arborea, Copenhageni, Icterohaemorrhagiae	RN, RR	[19, 127, 128]
Grenada	1	7.1%–24.5%	ND	Ballum, Cynopteri, Copenhageni, Icterohaemorrhagiae, Mankarso	RN	[129]
Guadeloupe (France)	1	32.0%	ND	Icterohaemorrhagiae	RN, RR	[19]
Puerto Rico (USA)	1	ND	0%–40.7%	ND	RN, RR, RAx, RFr	[130]
Trinidad (Trinidad and Tobago)	4	16.5%–20.5%	25.6%	Autumnalis, Ballum, Hebdomadis, Copenhageni, Icterohaemorrhagiae, Mankarso, Javanica, Louisiana	RN, RR	[19, 131–133]

Abbreviations: ND, no data; RAx, *R*. *alexandrinus*; RFr, *R*. *frugivorus*; RN, *R*. *norvegicus*; RR, *R*. *rattus*

**Table 8:** Summary of *Leptospira* prevalence in rats in South America.

Geographic location	Number of articles	Seroprevalence	Molecular/culture/ other prevalence	Serovars detected	Rat species	References
Argentina	3	41.8%–52.4%	2.4%–96.0%	Arborea, Castellonis, Canicola, Grippotyphosa, Hebdomadis, Icterohaemorrhagiae	RN, RR	[134–136]
Brazil	8	23.8%–100%	30.8%–91.7%	Australis, Autumnalis, Ballum, Castellonis, Whitcombi, Cynopteri, Djasiman, Sentot, Copenhageni, Icterohaemorrhagiae, Panama, Pyrogenes, Hardjo-minis, Hardjominiswajezak, Wolffi, Shermani, Tarassovi, Andamana, Patoc	RN	[137–145]
Chile	2	ND	19.6%–19.7%	ND	RN, RR	[146, 147]
Colombia	5	0%–25.2%	0%–48.6%	Australis, Bratislava, Ballum, Castellonis, Canicola, Grippotyphosa, Icterohaemorrhagiae, Pyrogenes, Hardjo, Sejroë, Shermani, Tarassovi, Valbuzzi	RN, RR	[148–152]
Ecuador	1	ND	3.0%	ND	ND	[153]
Peru	2	ND	9.8%–55.0%	Icterohaemorrhagiae, Varillal	RN, RR	[154, 155]

Abbreviations: ND, no data; RN, *R*. *norvegicus*; RR, *R*. *rattus*

The top five countries that were reported based on number of articles include India (*n* = 13), Malaysia (*n* = 9), Brazil (*n* = 8), Thailand (*n* = 7), and France (*n* = 6). Prevalence of *Leptospira* spp. in rats varied considerably. For example, two studies in Australia reported a very low prevalence of 1.7% [16] and 2.9% [15], and studies in China [31] and Ecuador [153] reported a similar prevalence of 3.0%. Several studies even reported zero prevalence of *Leptospira* spp. in rats, such as in Thailand [71], Madagascar [86], Tanzania [92], and the Faroe Islands [98]. Conversely, other studies reported a prevalence of more than 70%, such as in Brazil [137–139, 142, 145], Mexico [120], Egypt [82], Réunion [88], and the Philippines [58].

The prevalence reported from the same country had significant variations as well. For example, in Hawaii, four studies reported a prevalence of 16.0% [22], 24.4% [20], 30.2% [21], and 53.3% [19]. However, there were also instances in which independent studies from the same geographic location reported a similar prevalence, such as in Trinidad, with three studies which reported a prevalence of 16.5% [133], 17.4% [131], and 20.5% [19]. In Malaysia, of the seven articles retrieved, one reported a prevalence of 3.1% [51], whereas the rest had a similar prevalence between 8% and 18% [50, 53–57].

### Methods of detecting for *Leptospira* exposure or infection

A wide variety of diagnostic methods used were reported (Table 9). The most common diagnostic methods used were microscopic agglutination test (MAT), polymerase chain reaction (PCR), and culture and isolation. The majority of the studies (*n* = 90) used only one method of detection, either MAT, PCR, culture, or others.

**Table 9:** Distribution of methods of detecting *Leptospira* spp. used by all studies.

Method(s) of detection	Number of studies
Culture only	23
PCR only	31
MAT only	25
Others^a^ only	11
Culture and PCR	8
Culture and MAT	13
Culture and others^a^	9
PCR and MAT	8
PCR and others^a^	2
MAT and others^a^	1
Culture, PCR, and MAT	8
Culture, PCR, and others^a^	0
Culture, MAT, and others^a^	5
PCR, MAT, and others^a^	0
Culture, PCR, MAT, and others^a^	1
**Total**	145

^a^ Other methods include one or more of the following: ELISA, MSAT, CFT, IFAT, staining methods, IFA, DFA, DFM, IHC, and inoculation into laboratory animals.

Abbreviations: CFT, complement fixation test; DFA, direct immunofluorescence assay; DFM, dark-field microscopy; ELISA, enzyme-linked immunosorbent assay; IFA, indirect immunofluorescence assay; IFAT, indirect fluorescent antibody test; IHC, immunohistochemistry; MAT, microscopic agglutination test; MSAT, macroscopic slide agglutination test; PCR, polymerase chain reaction

Within the methods of PCR and culture, studies used several types of tissues and body fluids for the detection of *Leptospira* spp., with the most common being kidney and urine samples. Other samples utilized include blood, milk, liver, spleen, brain, lung, breast, and urinary bladder samples (Table 10). PCR target genes also varied, and the list of genes and the corresponding number of studies that used them can be found in Table 11. S2 Table illustrates all relevant data pertaining to diagnostic methods used, including the reported prevalence of *Leptospira* spp. in rats based on each specific method of detection.

**Table 10:** Distribution of samples used by studies that performed culture and/or PCR.

Method of detection	Number of studies
Culture of only kidney samples	48
Culture of only urine samples	3
Culture of kidney and urine samples	10
Culture of kidney and blood samples	1
Culture of kidney, urine, and blood samples	2
Culture of kidney, liver, and spleen samples	1
Culture of kidney, liver, and blood samples	1
Culture of kidney, liver, and brain samples	1
PCR of only kidney samples	38
PCR of only urine samples	2
PCR of only urinary bladder samples	1
PCR of only serum samples	3
PCR of kidney and urine samples	5
PCR of kidney and urinary bladder samples	1
PCR of kidney and spleen samples	1
PCR of kidney and lung samples	1
PCR of kidney, urine, and blood samples	1
PCR of kidney, liver, and spleen samples	1
PCR of kidney, liver, and blood samples	1
PCR of kidney, brain, and blood samples	1
PCR of kidney, breast, and milk samples	1
PCR of kidney, brain, blood, urinary bladder, and urine samples	1

Abbreviation: PCR, polymerase chain reaction

**Table 11:** PCR target genes used by 57 studies.

PCR target gene	Number of studies
*lipL32*	16
*lipL32* and *rrs* (16S rRNA)	4
*lipL32*, G1/G2 primers, and B64I/B64II primers	1
*rrs* (16S rRNA)	13
*rrs* (16S rRNA) and *hap1*	1
*rrs* (16S rRNA) and *secY*	1
*rrl* (23S rDNA)	1
*rrl* (23S rRNA) and LA0322	1
*hap1*	1
*secY*	4
*flaB*	3
G1/G2 primers	5
Lig1/Lig2 primers	1
TaqVet PathoLept Kit, LSI, Lissieu, France	3
***Leptospira*** PCR Kit, Shanghai ZJ Bio-Tech Co. Ltd, Shanghai, China	1
ND	1
**Total**	57

Abbreviations: LSI, Laboratoire Service International; ND, no data; PCR, polymerase chain reaction; rDNA, ribosomal deoxyribonucleic acid; rRNA, ribosomal ribonucleic acid

### Geographic distribution of serovars

A large number of serovars have been reported across all the studies retrieved, representing different geographic locations and continents. For each of those geographic locations, the serovars reported are listed in Tables 1–8. Studies conducted in Asia reported the highest number of different serovars detected (*n* = 30), followed by studies conducted in South America (*n* = 28). A comparison of serogroups and serovars detected in all represented countries can be found in S3 Table.

Interestingly, serovar Ballum has been reported in all represented countries in Oceania: Australia [16], Fiji [17], Hawaii [19–22], New Caledonia [23], and New Zealand [13, 24, 25], with the exception of Wallis and Futuna [26], which did not provide serovar information.

In Asia, frequently reported serovars include Icterohaemorrhagiae (reported in eight countries), Autumnalis, Javanica (reported in six countries), Australis, Canicola, Pomona, and Pyrogenes (reported in five countries). The most reported serovar in the Middle East was Icterohaemorrhagiae (reported in four countries), whereas the most reported serovar in Africa was Canicola (reported in three countries). In Europe, serovars Icterohaemorrhagiae and Sejroë were the most frequently reported serovars, detected in eight and three countries, respectively. Serovar Icterohaemorrhagiae was also the most frequently detected in North America, South America, and the Caribbean. Several serovars of the Icterohaemorrhagiae serogroup have been frequently reported in the Caribbean, including serovars Copenhageni, Icterohaemorrhagiae, and Mankarso. The most frequently reported serovar worldwide was Icterohaemorrhagiae, detected in 36 of 43 countries that provided serovar information.

### Prevalence of *Leptospira* spp. in various species of rats (*Rattus* spp.)

Among all rat species sampled in all studies, *R*. *norvegicus* and *R*. *rattus* were the two most frequently sampled species (Tables 1–8). Studies representing countries in Asia reported the most diverse species of rats sampled, with other species such as *R*. *exulans* and *R*. *argentiventer* in Cambodia, Malaysia, Thailand, Laos, and Vietnam; *R*. *tanezumi* in Cambodia, Laos, Thailand, and Vietnam; and *R*. *losea* in China, Cambodia, Laos, and Thailand (Table 2). Studies from Malaysia reported many other rat species that were not identified and sampled in other countries, such as *R*. *diardii*, *R*. *bowersi*, *R*. *muelleri*, *R*. *rajah*, *R*. *sabanus*, *R*. *tiomanicus*, and *R*. *whiteheadi*.

Overall, 30.3% (4,829/15,917) of *R*. *norvegicus*, 17.8% (2,376/13,353) of *R*. *rattus*, 10.9% (344/3,143) of *R*. *exulans*, 19.3% (87/451) of *R*. *argentiventer*, 3.4% (15/435) of *R*. *tanezumi*, and 13.1% (18/137) of *R*. *losea* were reported to be positive for *Leptospira* spp. In general, *R*. *norvegicus* was largely found to have a higher prevalence than *R*. *rattus* within the same studies. However, the opposite was also reported—i.e., the prevalence of *Leptospira* spp. in *R*. *rattus* was higher than *R*. *norvegicus* within the same studies. For example, on Réunion Island, one study reported a prevalence of 38.5% (214/562) in *R*. *rattus*, whereas *R*. *norvegicus* had a prevalence of 30.6% (52/170) [89]. The same was reported in New Zealand [13, 25], with *R*. *rattus* having higher prevalences (33.3%–34.4%) than *R*. *norvegicus* (25.7%–25.9%). The prevalence of *Leptospira* spp. in the various rat species still varied greatly among all the studies. Detailed information about *Leptospira* prevalence in each rat species per study can be found in S4 Table.

### Other studies involving laboratory or pet rats

We also found three studies pertaining to natural *Leptospira* infection in laboratory albino rat (*R*. *norvegicus*) colonies. Overall, relatively high prevalences were reported and could be a good discussion point. The three articles reported prevalences of 67.0% [156] (part 1), 26.9% [156] (part 2), 90.0% [157], and about 68.0% [158]. This demonstrated that *Leptospira* infection could even be endemic in laboratory colonies within controlled environments, most likely primarily caused by either carrier adult rats [156, 158] or infection from wild rats [158]. Most of the infections were caused by serovar Icterohaemorrhagiae [156, 158], but other serovars were also reported, such as serovar Javanica [157].

In addition, four European articles reported cases of human leptospirosis associated with transmission from pet rats, which were not included in this review. Two articles published in 2008 reported one case in 2006 in the UK [159] and another case in Germany (year not specified) [160]. *L*. *interrogans* serogroup Icterohaemorrhagiae was identified in both cases. An article published in 2012 in the Czech language reported three human patients treated for leptospirosis from 2005 to 2010 in Czech Republic, with likelihood that they acquired the infection from their pet rats [161]. Another article published in 2017 identified six human leptospirosis cases from 2009 to 2016 in Belgium and France, for which pet rodents were the source of infection [162]. Of the six cases, three were identified to be caused by serogroup Icterohaemorrhagiae and one by serogroup Sejroë.

## Discussion

With rapid global urbanization and 68% of the world’s human population projected to be living in urban areas by the year 2050 (55% as of 2018) [163], in addition to the increasing loss of natural habitats, it is anticipated that the majority of human–wildlife interactions would transpire in these areas. Wild rats seem to benefit from urbanization and thrive in urban and peridomestic environments, leading to frequent human exposure to these species [164]. *R*. *norvegicus* and *R*. *rattus* have become ubiquitous in urban environments and are significant sources of many zoonotic pathogens that can result in mortality and morbidity in humans and animals, and leptospirosis is one of those important rat-associated zoonoses. Because of knowledge gaps in the ecology of rats in urban environments, urban rat control is largely ineffective [165]; thus, it is critical to acquire a deeper understanding of the ecological and demographical drivers of zoonotic pathogen transmission in urban environments [164]. In addition, knowing the prevalence of a specific rat-associated zoonosis in a geographic region is essential in targeted pathogen screening in order to reduce underdiagnoses and misdiagnoses.

In a broad perspective, significant information has been documented in almost every country included in this literature review. However, the results reveal findings consistent with the common knowledge that incidence of leptospirosis is higher in tropical or warm-climate countries compared with countries in temperate regions. Of the top five countries represented in terms of number of articles, four of them are in tropical regions: India, Malaysia, Brazil, and Thailand. However, more studies representing tropical countries were retrieved compared with those from temperate regions. Many of the studies conducted also reported prevalences based on small sample sizes, which might not be indicative of the true distribution of *Leptospira* spp. in those geographic locations. This could be because of the convenience sampling of rats in conjunction with other rodents or animals; nevertheless, data pertaining to the rat samples were extracted for this review. In addition, the actual number of rats and population density in the wild may vary considerably among all geographic locations, which might indirectly affect the differences in prevalence of *Leptospira* spp. Differences in rat species predominating in a certain region may also potentially contribute to the differences in prevalence, in which further research is necessary for evaluation.

Several studies revealed zero prevalence of *Leptospira* spp. in the rats, which might be due to a number of possible reasons. For some of those studies, it could be simply due to the small sample size, with less than 50 rat samples obtained in studies in South Korea [61, 63], Thailand [71], Mayotte [19], Canada [116], Austria [94], and Hungary [94]. Another reason could be due to climatic conditions, which resulted in zero prevalence reported in a study in the Faroe Islands, to which they concluded that it could either be too cold for *Leptospira* transmission or too cold for the maintenance of adequate densities of rats [98]. An article from Madagascar reported a prevalence of 0% despite having a relatively decent sample size, and it concluded that *Leptospira* spp. was likely not present in Madagascar [86]. However, a more recent article reported a prevalence of 40.0% [85], which could indicate a relatively recent change in geographical distribution of *Leptospira* spp. in Madagascar or that the methodology used in the earlier study was not optimal. A study done in Tanzania revealed zero prevalence in 384 rodents (including 320 rats), which is a notable finding, considering the rodents were sampled in areas known to have a high incidence of human leptospirosis. This might indicate that peridomestic rodents are not a major source of human infection in that area [92].

Prevalences reported by studies from the same country also varied, which could be because of reasons such as differences in methodology or sampling from different parts of that country. As seen in the Results section, methods of detecting for *Leptospira* spp. varied greatly among all the studies retrieved, which may have certain implications on detection sensitivity. Some diagnostic methods such as PCR and other molecular methods detect *Leptospira* nucleic acids, whereas serological methods such as MAT detect anti-*Leptospira* antibodies, which only indicates exposure to the bacterium and not necessarily a current infection. Moreover, such serological techniques possess a risk of cross-reaction; thus, their results should be interpreted with caution. PCR protocols, in general, are used for detecting pathogenic *Leptospira* spp., and isolation of infecting *Leptospira* strains followed by cumbersome serological methods are required for identifying the serovars, which is not pursued in many studies reported. In culture methods with the usage of dark-field microscopy for identification of spirochetes, there may be possibilities of human error or issues regarding ambiguity of results as well, with possible false positives or negatives. In addition, varied tissue samples were used among the publications retrieved, and some studies used only one type of tissue sample, whereas others used multiple types of samples, pooled or unpooled. There are also different sensitivities and specificities for each of the various diagnostic methods. All these factors could affect the detection of *Leptospira* spp., which may have contributed to the varied prevalences observed.

The diversity of serovars detected varied considerably among studies and geographic regions. This review revealed that studies conducted in Asia and South America detected the highest number of different serovars, which could have been influenced by several factors. Methodologies in general could influence this, with different methods of serovar characterization being used among the studies. In seroprevalence studies that used MAT, serovars not included in the diagnostic panel would not be detected and, thus, affect the study results. With Asia and South America being tropical regions, there could be plausible correlations with the large variety of serovars present. Factors to be considered are the higher incidence of *Leptospira* spp. in tropical regions and the relatively higher number of studies retrieved from Asia and South America, which could provide higher serovar diversity.

Several possible correlations with regard to geographic distribution of infecting serovars could be observed. Serovar Ballum could be the main infecting serovar in rats in Oceania, with all studies that provided serovar information having identified the presence of serovar Ballum. Serovar Sejroë was reported in European countries more than countries from other regions. Interestingly, most of the studies conducted in the Caribbean concurrently identified several serovars of the Icterohaemorrhagiae serogroup (serovars Copenhageni, Icterohaemorrhagiae, and Mankarso) more frequently than studies in other geographic regions. Overall, serovar Icterohaemorrhagiae was the most frequently reported serovar, identified in almost all represented geographic locations. In countries that did not report serovar Icterohaemorrhagiae, certain methodological factors discussed earlier in this section could be the reason for the absence. For example, in Australia, despite retrieving three articles, only one provided serovar information. However, the extremely low prevalence (1.7%) reported in that article meant that the true variety of serovars present may have been underestimated.

Comparing the two most common rat species sampled, reported *Leptospira* prevalence was generally higher in *R*. *norvegicus* than *R*. *rattus*. This may suggest a possible correlation with the species of rats and susceptibility of *Leptospira* infection. However, many of the studies use morphological characteristics and general appearance to determine the exact rat species and, thus, may cause inaccurate reporting of results. For a number of studies, rat species were not determined and reported, which may have certain implications. As there are many other species of small mammals and rodents that resemble rats, they may not be of the genus *Rattus* and, therefore, are not considered “true” rats. The taxonomy of rats has also changed throughout the years, and rats that were once known to be under the *Rattus* genus may not be classified as one in the present day. Twleve species reported by studies have been identified to be potentially not of the *Rattus* genus or are subspecies of *R*. *rattus* (S5 Table) [166, 167]. If such small mammals and rodents were misidentified as rats of the *Rattus* genus and reported in studies, this could be another reason for inaccurate reporting of results. In addition, some studies also combined the results of all rodents sampled, including mice and other rodents, and did not provide separate data and results for each type of animal. This caused inconsistencies in extraction of data from those articles. For future studies, inclusion of all information such as exact species of rats and the individual prevalence of each of those species will lower the inconsistency.

Our review also identified relatively high prevalences of *Leptospira* spp. in laboratory rat colonies within controlled environments, indicating primary carrier status in laboratory rats or inadvertent transmission and spillover infection from wild rats. In addition to possible interference with biomedical research, one of the main implications of high *Leptospira* prevalence in laboratory rat colonies is the zoonotic potential to laboratory animal caretakers, as evidenced by Natrajaseenivasan and Ratnam [157], who reported a very high seropositivity (91.0%) in animal house workers.

Several cases of human leptospirosis associated with transmission from pet rats demonstrate that wild rats are not the only sources of rodent-associated human infection. With the rising popularity of keeping rats as household pets, there may be concerns about pet rats being a potential source of human *Leptospira* infection, and exposure to infected pet rats could pose a significant public health risk.

## Conclusions and recommendations

This review summarizes the literature on global prevalence and distribution of *Leptospira* infection in rats. Prevalence of *Leptospira* spp. varies widely, with a considerably high prevalence reported in many countries involving multiple rat species. This review also demonstrated several weaknesses to the current methods of detecting and documenting *Leptospira* prevalence in rats worldwide. As such, this necessitates a call for standardized protocols for the detection and reporting of such studies, especially pertaining to the diagnostic methods used. In addition, appropriate quality control programs using standardized region-specific diagnostic panels, as well as improvements in techniques for serovar differentiation, could be proposed. A deeper understanding of the ecology and epidemiology of *Leptospira* spp. in rats in urban environments is warranted. It is also pertinent for rat control programs to be implemented in conjunction with increased efforts for public awareness and education regarding leptospirosis transmission and prevention.

**Key learning points**

- Prevalence of *Leptospira* infection in rats varies widely, with a considerably high prevalence reported in many countries involving multiple rat species.
- *Leptospira* prevalence in rats is higher in geographical regions with tropical climates compared with regions with temperate climates.
- Serovar Icterohaemorrhagiae is the most prevalent serovar reported in rats.
- Weaknesses to the current methods of detecting and documenting *Leptospira* prevalence in rats necessitates a call for standardized protocols for reporting in such studies.
- It is pertinent for rat control programs to be implemented in conjunction with increased efforts for public awareness and education regarding leptospirosis transmission and prevention.

**Top five papers**

1. Desvars A, Cardinale E, Michault A. Animal leptospirosis in small tropical areas. Epidemiol Infect. 2011;139(2):167–88. Epub 2010/09/30. doi: 10.1017/S0950268810002074. PubMed PMID: 20875197.
2. Hathaway SC, Blackmore DK. Ecological aspects of the epidemiology of infection with leptospires of the Ballum serogroup in the black rat (Rattus rattus) and the brown rat (Rattus norvegicus) in New Zealand. J Hyg (Lond). 1981;87(3):427–36. Epub 1981/12/01. PubMed PMID: 7310125; PubMed Central PMCID: PMCPMC2134120.
3. de Faria MT, Calderwood MS, Athanazio DA, McBride AJ, Hartskeerl RA, Pereira MM, et al. Carriage of Leptospira interrogans among domestic rats from an urban setting highly endemic for leptospirosis in Brazil. Acta Trop. 2008;108(1):1–5. Epub 2008/08/30. doi: 10.1016/j.actatropica.2008.07.005. PubMed PMID: 18721789; PubMed Central PMCID: PMCPMC2596941.
4. Himsworth CG, Jardine CM, Parsons KL, Feng AYT, Patrick DM. The Characteristics of Wild Rat (Rattus spp.) Populations from an Inner-City Neighborhood with a Focus on Factors Critical to the Understanding of Rat-Associated Zoonoses. PLoS ONE. 2014;9(3):e91654. doi: 10.1371/journal.pone.0091654.
5. Ellis WA. Animal Leptospirosis. In: Adler B, editor. Leptospira and Leptospirosis. Berlin, Heidelberg: Springer Berlin Heidelberg; 2015. p. 99–137.

## Supporting information

S1 Table Summary of global *Leptospira* prevalence and serovar distribution. (XLSX)

S2 Table Summary of *Leptospira* prevalence by detection methods. (XLSX)

S3 Table Distribution of *Leptospira* serovars reported, sorted by continents. (XLSX)

S4 Table Summary of *Leptospira* prevalence by species of rats. (XLSX)

S5 Table Taxonomic synonymy of rat species reported in studies. (DOCX)

S1 List List of all 145 publications included in the literature review. (DOCX)

S1 Map Interactive map of the geographic distribution of all publications included in the literature review. (HTML)
