# Supplementary material for: Leptospira infection in rats: A literature review of global prevalence and distribution
Source: PLoS Negl Trop Dis. 2019 Aug 9;13(8):e0007499. doi: 10.1371/journal.pntd.0007499 (PMC6688788; doi:10.1371/journal.pntd.0007499)
Supplement: S5 Table — (DOCX) [file pntd.0007499.s005.docx]

**S5 Table. Taxonomic synonymy of rat species reported in studies.**

| Taxonomic synonym reported | Rodent species | Genbank common name |
| --- | --- | --- |
| *Rattus alexandrinus* | *Rattus rattus alexandrinus* | Black rat (subspecies) |
| *Rattus bowersi* | *Berylmys bowersi* | Bower’s white-toothed rat |
| *Rattus diardii* | *Rattus rattus diardii* | Malayan black rat |
| *Rattus frugivorus* | *Rattus rattus frugivorus* | Black rat (subspecies) |
| *Rattus fulvescens* | *Niniventer fulvescens* | Chestnut white-bellied rat |
| *Rattus hinton* | *Rattus rattus wroughtoni* | Black rat (subspecies) |
| *Rattus muelleri* | *Sundamys muelleri* | Muller’s giant Sunda rat |
| *Rattus rufescens* | *Rattus rattus rufescens* | Black rat (subspecies) |
| *Rattus rajah* | *Maxomys rajah* | Rajah spiny rat |
| *Rattus sabanus* | *Leopoldamys sabanus* | Long-tailed giant rat |
| *Rattus surifer* | *Maxomys surifer* | Indomalayan maxomys |
| *Rattus whiteheadi* | *Maxomys whiteheadi* | Whitehead’s spiny rat |
