# Supplementary material for: Leptospira infection in rats: A literature review of global prevalence and distribution
Source: PLoS Negl Trop Dis. 2019 Aug 9;13(8):e0007499. doi: 10.1371/journal.pntd.0007499 (PMC6688788; doi:10.1371/journal.pntd.0007499)
Supplement: S1 List — (DOCX) [file pntd.0007499.s006.docx]

**S1 List. List of all 145 publications included in the literature review.**

1. Agudelo-Florez, P., Arango, J. C., Merizalde, E., Londono, A. F., Quiroz, V. H., & Rodas, J. D. (2010). Serological evidence of Leptospira spp circulation in naturally-exposed rats (Rattusnorvegicus) in a Colombian urban area. *Rev Salud Publica (Bogota), 12*(6), 990-999.

2. Agudelo-Florez, P., Londono, A. F., Quiroz, V. H., Angel, J. C., Moreno, N., Loaiza, E. T., . . . Rodas, J. D. (2009). Prevalence of Leptospira spp. in urban rodents from a groceries trade center of Medellin, Colombia. *Am J Trop Med Hyg, 81*(5), 906-910. doi:10.4269/ajtmh.2009.09-0195

3. Allan, K. J., Halliday, J. E. B., Moseley, M., Carter, R. W., Ahmed, A., Goris, M. G. A., . . . Cleaveland, S. (2018). Assessment of animal hosts of pathogenic Leptospira in northern Tanzania. *PLoS Negl Trop Dis, 12*(6), e0006444. doi:10.1371/journal.pntd.0006444

4. Allen, S. E., Ojkic, D., & Jardine, C. M. (2014). Prevalence of antibodies to Leptospira in wild mammals trapped on livestock farms in Ontario, Canada. *J Wildl Dis, 50*(3), 666-670. doi:10.7589/2013-11-292

5. Amaddeo, D., Ieradi, L. A., Autorino, G. L., & Perrella, D. (1996). Leptospirosis in wild rodents living in urban areas (Rome - Italy). *Proceedings of the I European Congress of Manvnalogy*, 105·114.

6. Assenga, J. A., Matemba, L. E., Muller, S. K., Mhamphi, G. G., & Kazwala, R. R. (2015). Predominant leptospiral serogroups circulating among humans, livestock and wildlife in Katavi-Rukwa ecosystem, Tanzania. *PLoS Negl Trop Dis, 9*(3), e0003607. doi:10.1371/journal.pntd.0003607

7. Aviat, F., Blanchard, B., Michel, V., Blanchet, B., Branger, C., Hars, J., . . . Andre-Fontaine, G. (2009). Leptospira exposure in the human environment in France: A survey in feral rodents and in fresh water. *Comp Immunol Microbiol Infect Dis, 32*(6), 463-476. doi:10.1016/j.cimid.2008.05.004

8. Ayral, F., Artois, J., Zilber, A. L., Widen, F., Pounder, K. C., Aubert, D., . . . Artois, M. (2015a). The relationship between socioeconomic indices and potentially zoonotic pathogens carried by wild Norway rats: a survey in Rhone, France (2010-2012). *Epidemiol Infect, 143*(3), 586-599. doi:10.1017/S0950268814001137

9. Ayral, F., Zilber, A. L., Bicout, D. J., Kodjo, A., Artois, M., & Djelouadji, Z. (2015b). Distribution of Leptospira interrogans by Multispacer Sequence Typing in Urban Norway Rats (Rattus norvegicus): A Survey in France in 2011-2013. *PLoS One, 10*(10), e0139604. doi:10.1371/journal.pone.0139604

10. Barragan, V., Chiriboga, J., Miller, E., Olivas, S., Birdsell, D., Hepp, C., . . . Pearson, T. (2016). High Leptospira Diversity in Animals and Humans Complicates the Search for Common Reservoirs of Human Disease in Rural Ecuador. *PLoS Negl Trop Dis, 10*(9), e0004990. doi:10.1371/journal.pntd.0004990\

11. Benacer, D., Mohd Zain, S. N., Amran, F., Galloway, R. L., & Thong, K. L. (2013). Isolation and molecular characterization of Leptospira interrogans and Leptospira borgpetersenii isolates from the urban rat populations of Kuala Lumpur, Malaysia. *Am J Trop Med Hyg, 88*(4), 704-709. doi:10.4269/ajtmh.12-0662

12. Benacer, D., Mohd Zain, S. N., Sim, S. Z., Mohd Khalid, M. K., Galloway, R. L., Souris, M., & Thong, K. L. (2016). Determination of Leptospira borgpetersenii serovar Javanica and Leptospira interrogans serovar Bataviae as the persistent Leptospira serovars circulating in the urban rat populations in Peninsular Malaysia. *Parasit Vectors, 9*, 117. doi:10.1186/s13071-016-1400-1

13. Bezjak, V., & Thorburn, H. (1983). Survey of rats (Rattus norvegicus) in Kuwait for the presence of Leptospira. *Trop Geogr Med, 35*(1), 33-36.

14. Biscornet, L., Dellagi, K., Pages, F., Bibi, J., de Comarmond, J., Melade, J., . . . Tortosa, P. (2017). Human leptospirosis in Seychelles: A prospective study confirms the heavy burden of the disease but suggests that rats are not the main reservoir. *PLoS Negl Trop Dis, 11*(8), e0005831. doi:10.1371/journal.pntd.0005831

15. Bojiraj, M., Porteen, K., Gunaseelan, L., & Sureshkannan, S. (2017). Seroprevalence of Leptospirosis in Animals and Its Public Health Significance. *International Journal of Livestock Research, 7*(11), 220-226. doi:http://dx.doi.org/10.5455/ijlr.20170812041853

16. Broom, J. C., & Gibson, E. A. (1953). Infection rates of Rattus norvegicus with Leptospira icterohaemorrhagiae in Great Britain. I. A rural area in Carmarthenshire, Wales. *J Hyg (Lond), 51*(3), 416-425.

17. Calderón, A., Rodriguez, V., Mattar, S., & Arrieta, G. (2014). Leptospirosis in pigs, dogs, rodents, humans, and water in an area of the Colombian tropics. *Trop Anim Health Prod, 46*(2), 427-432. doi:10.1007/s11250-013-0508-y

18. Carter, M. E., & Cordes, D. O. (1980). Leptospirosis and other infections of Battus rattus and Rattus norvegicus. *N Z Vet J, 28*(3), 45-50. doi:10.1080/00480169.1980.34688

19. Chakma, S., Picard, J., Duffy, R., Constantinoiu, C., & Gummow, B. (2017). A Survey of Zoonotic Pathogens Carried by Non-Indigenous Rodents at the Interface of the Wet Tropics of North Queensland, Australia. *Transbound Emerg Dis, 64*(1), 185-193. doi:10.1111/tbed.12360

20. Collares-Pereira, M., Korver, H., Terpstra, W. J., Santos-Reis, M., Ramalhinho, M. G., Mathias, M. L., . . . Petrucci-Fonseca, F. (1997). First epidemiological data on pathogenic leptospires isolated on the Azorean islands. *Eur J Epidemiol, 13*(4), 435-441.

21. Collares-Pereira, M., Mathias, M. L., Santos-Reis, M., Ramalhinho, M. G., & Duarte-Rodrigues, P. (2000). Rodents and Leptospira transmission risk in Terceira island (Azores). *Eur J Epidemiol, 16*(12), 1151-1157.

22. Collings, D. F. (1984). Leptospira interrogans infection in domestic and wild animals in Fiji. *N Z Vet J, 32*(3), 21-24. doi:10.1080/00480169.1984.35050

23. Correa, J. P., Bucarey, S. A., Cattan, P. E., Landaeta-Aqueveque, C., & Ramirez-Estrada, J. (2017). Renal carriage of Leptospira species in rodents from Mediterranean Chile: The Norway rat (Rattus norvegicus) as a relevant host in agricultural lands. *Acta Trop, 176*, 105-108. doi:10.1016/j.actatropica.2017.07.032

24. Cosson, J. F., Picardeau, M., Mielcarek, M., Tatard, C., Chaval, Y., Suputtamongkol, Y., . . . Morand, S. (2014). Epidemiology of Leptospira transmitted by rodents in Southeast Asia. *PLoS Negl Trop Dis, 8*(6), e2902. doi:10.1371/journal.pntd.0002902

25. Costa, F., Porter, F. H., Rodrigues, G., Farias, H., de Faria, M. T., Wunder, E. A., . . . Childs, J. E. (2014). Infections by Leptospira interrogans, Seoul virus, and Bartonella spp. among Norway rats (Rattus norvegicus) from the urban slum environment in Brazil. *Vector Borne Zoonotic Dis, 14*(1), 33-40. doi:10.1089/vbz.2013.1378

26. de Faria, M. T., Calderwood, M. S., Athanazio, D. A., McBride, A. J., Hartskeerl, R. A., Pereira, M. M., . . . Reis, M. G. (2008). Carriage of Leptospira interrogans among domestic rats from an urban setting highly endemic for leptospirosis in Brazil. *Acta Trop, 108*(1), 1-5. doi:10.1016/j.actatropica.2008.07.005

27. de Oliveira, D., Figueira, C. P., Zhan, L., Pertile, A. C., Pedra, G. G., Gusmao, I. M., . . . Costa, F. (2016). Leptospira in breast tissue and milk of urban Norway rats (Rattus norvegicus). *Epidemiol Infect, 144*(11), 2420-2429. doi:10.1017/S0950268816000637

28. Della Rossa, P., Tantrakarnapa, K., Sutdan, D., Kasetsinsombat, K., Cosson, J. F., Supputamongkol, Y., . . . Morand, S. (2016). Environmental factors and public health policy associated with human and rodent infection by leptospirosis: a land cover-based study in Nan province, Thailand. *Epidemiol Infect, 144*(7), 1550-1562. doi:10.1017/s0950268815002903

29. Denipitiya, D. T., Chandrasekharan, N. V., Abeyewickreme, W., Hartskeerl, R. A., & Hapugoda, M. D. (2017). Identification of cattle, buffaloes and rodents as reservoir animals of Leptospira in the District of Gampaha, Sri Lanka. *BMC Res Notes, 10*(1), 134. doi:10.1186/s13104-017-2457-4

30. Desvars, A., Cardinale, E., & Michault, A. (2011). Animal leptospirosis in small tropical areas. *Epidemiol Infect, 139*(2), 167-188. doi:10.1017/S0950268810002074

31. Desvars, A., Naze, F., Benneveau, A., Cardinale, E., & Michault, A. (2013). Endemicity of leptospirosis in domestic and wild animal species from Reunion Island (Indian Ocean). *Epidemiol Infect, 141*(6), 1154-1165. doi:10.1017/S0950268812002075

32. Desvars-Larrive, A., Pascal, M., Gasqui, P., Cosson, J. F., Benoit, E., Lattard, V., . . . Vourc'h, G. (2017). Population genetics, community of parasites, and resistance to rodenticides in an urban brown rat (Rattus norvegicus) population. *PLoS One, 12*(9), e0184015. doi:10.1371/journal.pone.0184015

33. Doungchawee, G., Phulsuksombat, D., Naigowit, P., Khoaprasert, Y., Sangjun, N., Kongtim, S., & Smythe, L. (2005). Survey of leptospirosis of small mammals in Thailand.

34. Dybing, N. A., Jacobson, C., Irwin, P., Algar, D., & Adams, P. J. (2017). Leptospira Species in Feral Cats and Black Rats from Western Australia and Christmas Island. *Vector Borne Zoonotic Dis, 17*(5), 319-324. doi:10.1089/vbz.2016.1992

35. Easterbrook, J. D., Kaplan, J. B., Vanasco, N. B., Reeves, W. K., Purcell, R. H., Kosoy, M. Y., . . . Klein, S. L. (2007). A survey of zoonotic pathogens carried by Norway rats in Baltimore, Maryland, USA. *Epidemiol Infect, 135*(7), 1192-1199. doi:10.1017/S0950268806007746

36. Esfandiari, B., Pourshafie, M. R., Gouya, M. M., Khaki, P., Mostafavi, E., Darvish, J., . . . Nahrevanian, H. (2015). An epidemiological comparative study on diagnosis of rodent leptospirosis in Mazandaran Province, northern Iran. *Epidemiol Health, 37*, e2015012. doi:10.4178/epih/e2015012

37. Everard, C. O., Fraser-Chanpong, G. M., Bhagwandin, L. J., Race, M. W., & James, A. C. (1983). Leptospires in wildlife from Trinidad and Grenada. *J Wildl Dis, 19*(3), 192-199.

38. Ferreira, A. S., Costa, P., Rocha, T., Amaro, A., Vieira, M. L., Ahmed, A., . . . Inacio, J. (2014). Direct detection and differentiation of pathogenic Leptospira species using a multi-gene targeted real time PCR approach. *PLoS One, 9*(11), e112312. doi:10.1371/journal.pone.0112312

39. Firth, C., Bhat, M., Firth, M. A., Williams, S. H., Frye, M. J., Simmonds, P., . . . Lipkin, W. I. (2014). Detection of zoonotic pathogens and characterization of novel viruses carried by commensal Rattus norvegicus in New York City. *MBio, 5*(5), e01933-01914. doi:10.1128/mBio.01933-14

40. Foronda, P., Martin-Alonso, A., Del Castillo-Figueruelo, B., Feliu, C., Gil, H., & Valladares, B. (2011). Pathogenic Leptospira spp. in wild rodents, Canary Islands, Spain. *Emerg Infect Dis, 17*(9), 1781-1782. doi:10.3201/eid1709.101470

41. Freulon, M., Aboubaker, M., Marie, J. L., Drancourt, M., & Davoust, B. (2010). Detection of Leptospira organisms in Rattus rattus of two islands in the Mozambique Channel: Europa and Juan-de-Nova. *Bull Soc Pathol Exot, 103*(1), 48-50. doi:10.1007/s13149-009-0036-1

42. Gamage, C. D., Koizumi, N., Muto, M., Nwafor-Okoli, C., Kurukurusuriya, S., Rajapakse, J. R., . . . Tamashiro, H. (2011). Prevalence and carrier status of leptospirosis in smallholder dairy cattle and peridomestic rodents in Kandy, Sri Lanka. *Vector Borne Zoonotic Dis, 11*(8), 1041-1047. doi:10.1089/vbz.2010.0153

43. Gangadhar, N. L., Rajasekhar, M., Smythe, L. D., Norris, M. A., Symonds, M. L., & Dohnt, M. F. (2000). Reservoir hosts of Leptospira inadai in India. *Rev Sci Tech, 19*(3), 793-799.

44. Guernier, V., Lagadec, E., Cordonin, C., Le Minter, G., Gomard, Y., Pages, F., . . . Dellagi, K. (2016). Human Leptospirosis on Reunion Island, Indian Ocean: Are Rodents the (Only) Ones to Blame? *PLoS Negl Trop Dis, 10*(6), e0004733. doi:10.1371/journal.pntd.0004733

45. Guernier, V., Richard, V., Nhan, T., Rouault, E., Tessier, A., & Musso, D. (2017). Leptospira diversity in animals and humans in Tahiti, French Polynesia. *PLoS Negl Trop Dis, 11*(6), e0005676. doi:10.1371/journal.pntd.0005676

46. Halliday, J. E., Knobel, D. L., Allan, K. J., de, C. B. B. M., Handel, I., Agwanda, B., . . . Breiman, R. F. (2013). Urban leptospirosis in Africa: a cross-sectional survey of Leptospira infection in rodents in the Kibera urban settlement, Nairobi, Kenya. *Am J Trop Med Hyg, 89*(6), 1095-1102. doi:10.4269/ajtmh.13-0415

47. Hathaway, S. C., & Blackmore, D. K. (1981). Ecological aspects of the epidemiology of infection with leptospires of the Ballum serogroup in the black rat (Rattus rattus) and the brown rat (Rattus norvegicus) in New Zealand. *J Hyg (Lond), 87*(3), 427-436.

48. Hathaway, S. C., Blackmore, D. K., & Marshall, R. B. (1981). Leptospirosis in free-living species in New Zealand. *J Wildl Dis, 17*(4), 489-496.

49. Heuser, E., Fischer, S., Ryll, R., Mayer-Scholl, A., Hoffmann, D., Spahr, C., . . . Ulrich, R. G. (2017). Survey for zoonotic pathogens in Norway rat populations from Europe. *Pest Manag Sci, 73*(2), 341-348. doi:10.1002/ps.4339

50. Higa, H. H., & Fujinaka, I. T. (1976). Prevalence of rodent and mongoose leptospirosis on the Island of Oahu. *Public Health Rep, 91*(2), 171-177.

51. Himsworth, C. G., Bidulka, J., Parsons, K. L., Feng, A. Y., Tang, P., Jardine, C. M., . . . Patrick, D. M. (2013). Ecology of Leptospira interrogans in Norway rats (Rattus norvegicus) in an inner-city neighborhood of Vancouver, Canada. *PLoS Negl Trop Dis, 7*(6), e2270. doi:10.1371/journal.pntd.0002270

52. Ivanova, S., Herbreteau, V., Blasdell, K., Chaval, Y., Buchy, P., Guillard, B., & Morand, S. (2012). Leptospira and rodents in Cambodia: environmental determinants of infection. *Am J Trop Med Hyg, 86*(6), 1032-1038. doi:10.4269/ajtmh.2012.11-0349

53. Jensen, P. M., & Magnussen, E. (2016). Is it too cold for Leptospira interrrogans transmission on the Faroese Islands? *Infect Dis (Lond), 48*(2), 156-160. doi:10.3109/23744235.2015.1092579

54. Jittimanee, J., & Wongbutdee, J. (2014). Survey of pathogenic Leptospira in rats by polymerase chain reaction in Sisaket Province. *J Med Assoc Thai, 97 Suppl 4*, S20-24.

55. Johnson, M. A., Smith, H., Joeph, P., Gilman, R. H., Bautista, C. T., Campos, K. J., . . . Vinetz, J. M. (2004). Environmental exposure and leptospirosis, Peru. *Emerg Infect Dis, 10*(6), 1016-1022. doi:10.3201/eid1006.030660

56. Kalfayan, B. H. (1947). Leptospira icterohaemorrhagiae in rats of Beirut. *Trans R Soc Trop Med Hyg, 40*(6), 895-900.

57. Kawabata, H., Sakakibara, S., Imai, Y., Masuzawa, T., Fujita, H., Tsurumi, M., . . . Watanabe, H. (2006). First record of Leptospira borgpetersenii isolation in the Amami Islands, Japan. *Microbiol Immunol, 50*(6), 429-434.

58. Keenan, J., Sharma, R., Dicker, R., Rayner, J., & Stone, D. (2009). Seroprevalence of Leptospira in rattus norvegicus in Grenada, West Indies. *West Indian Med J, 58*(2), 114-117.

59. Khairani-Bejo, S., Oii, S. S., & Bahaman, A. R. (2004). Rats: Leptospirosis Reservoir in Serdang Selangor Residential Area. *Journal of Animal and Veterinary Advances, 3*(2), 66-69.

60. Kim, H. C., Klein, T. A., Chong, S. T., Collier, B. W., Usa, M., Yi, S. C., . . . Song, J. W. (2007). Seroepidemiological survey of rodents collected at a U.S. military installation, Yongsan Garrison, Seoul, Republic of Korea. *Mil Med, 172*(7), 759-764.

61. Kobayashi, Y., Kusaba, T., & Ueki, R. (1972). Isolation of Leptospira javanica from rats on Ishigaki Island. *Am J Trop Med Hyg, 21*(3), 342-344. doi:10.4269/ajtmh.1972.21.342

62. Koizumi, N., Muto, M., Tanikawa, T., Mizutani, H., Sohmura, Y., Hayashi, E., . . . Watanabe, H. (2009). Human leptospirosis cases and the prevalence of rats harbouring Leptospira interrogans in urban areas of Tokyo, Japan. *J Med Microbiol, 58*(Pt 9), 1227-1230. doi:10.1099/jmm.0.011528-0

63. Koma, T., Yoshimatsu, K., Yasuda, S. P., Li, T., Amada, T., Shimizu, K., . . . Arikawa, J. (2013). A survey of rodent-borne pathogens carried by wild Rattus spp. in Northern Vietnam. *Epidemiol Infect, 141*(9), 1876-1884. doi:10.1017/S0950268812002385

64. Kositanont, U., Naigowit, P., Imvithaya, A., Singchai, C., & Puthavathana, P. (2003). Prevalence of antibodies to Leptospira serovars in rodents and shrews trapped in low and high endemic areas in Thailand. *J Med Assoc Thai, 86*(2), 136-142.

65. Koteeswaran, A. (2006). Seroprevalence of leptospirosis in man and animals in Tamilnadu. *Indian J Med Microbiol, 24*(4), 329-331.

66. Krøjgaard, L. H., Villumsen, S., Markussen, M. D., Jensen, J. S., Leirs, H., & Heiberg, A. C. (2009). High prevalence of Leptospira spp. in sewer rats (Rattus norvegicus). *Epidemiol Infect, 137*(11), 1586-1592. doi:10.1017/S0950268809002647

67. Kudo, Y., Vansith, K., Rin, E., Uchida, K., Kodama, S., Fukui, T., . . . Masuzawa, T. (2018). Molecular Epidemiological Survey of Leptospira Infection of Wild Rodents in the Urban Settlement of Cambodia. *Vector Borne Zoonotic Dis, 18*(3), 144-150. doi:10.1089/vbz.2017.2198

68. Kuriakose, M., Paul, R., Joseph, M. R., Sugathan, S., & Sudha, T. N. (2008). Leptospirosis in a midland rural area of Kerala State. *Indian J Med Res, 128*(3), 307-312.

69. Lagadec, E., Gomard, Y., Le Minter, G., Cordonin, C., Cardinale, E., Ramasindrazana, B., . . . Dellagi, K. (2016). Identification of Tenrec ecaudatus, a Wild Mammal Introduced to Mayotte Island, as a Reservoir of the Newly Identified Human Pathogenic Leptospira mayottensis. *PLoS Negl Trop Dis, 10*(8), e0004933. doi:10.1371/journal.pntd.0004933

70. Lahiri, M. N. (1941). Studies on Leptospira Icterohaemorrhagiae in Rats in Bombay City. *Ind Med Gaz, 76*(9), 536-538.

71. Latifah, I., Rahmat, M. S., Hayarti, K. B., Paramasvaran, S., Azizah, M. R., Imran, F., & Normaznah, Y. (2012). Prevalence of leptospiral DNA among wild rodents from a selected area in Beguk Dam Labis, Segamat, Johor, Malaysia. *Malays J Pathol, 34*(2), 157-159.

72. Latifah, I., Abdul Halim, A., Rahmat, M. S., Nadia, M. F., Ubil, Z. E., Asmah, H., . . . Nasir, M. A. (2017). Isolation by culture and PCR identification of LipL32 gene of pathogenic Leptospira spp. in wild rats of Kuala Lumpur. *Malays J Pathol, 39*(2), 161-166.

73. Levett, P. N., Walton, D., Waterman, L. D., Whittington, C. U., Mathison, G. E., & Everard, C. O. (1998). Surveillance of leptospiral carriage by feral rats in Barbados. *West Indian Med J., 47*(1), 15-17.

74. Li, H. Y., & Davis, D. E. (1952). The prevalence of carriers of Leptospira and Salmonella in Norway rats of Baltimore. *Am J Hyg, 56*(1), 90-91.

75. Li, S., Wang, D., Zhang, C., Wei, X., Tian, K., Li, X., . . . Yan, J. (2013). Source tracking of human leptospirosis: serotyping and genotyping of Leptospira isolated from rodents in the epidemic area of Guizhou province, China. *BMC Microbiol, 13*, 75. doi:10.1186/1471-2180-13-75

76. Lilenbaum, W., Ribeiro, V., Martin, E., & Bispo, V. (1993). Serologic study for detecting anti-Leptospira antibodies in Rattus norvegicus from Duque de Caxias, Rio de Janeiro, Brazil. *Rev Latinoam Microbiol, 35*(4), 357-380.

77. Lindenbaum, I., & Eylan, E. (1982). Leptospirosis in Rattus norvegicus and Rattus rattus in Israel. *Isr J Med Sci, 18*(2), 271-275.

78. Loan, H. K., Van Cuong, N., Takhampunya, R., Kiet, B. T., Campbell, J., Them, L. N., . . . Carrique-Mas, J. J. (2015). How important are rats as vectors of leptospirosis in the Mekong Delta of Vietnam? *Vector Borne Zoonotic Dis, 15*(1), 56-64. doi:10.1089/vbz.2014.1613

79. Martins, G., & Lilenbaum, W. (2013). The panorama of animal leptospirosis in Rio de Janeiro, Brazil, regarding the seroepidemiology of the infection in tropical regions. *BMC Vet Res, 9*, 237. doi:10.1186/1746-6148-9-237

80. Matthias, M. A., Ricaldi, J. N., Cespedes, M., Diaz, M. M., Galloway, R. L., Saito, M., . . . Vinetz, J. M. (2008). Human leptospirosis caused by a new, antigenically unique Leptospira associated with a Rattus species reservoir in the Peruvian Amazon. *PLoS Negl Trop Dis, 2*(4), e213. doi:10.1371/journal.pntd.0000213

81. McKiel, J. A., Cousineau, J. G., & Hall, R. R. (1961). Leptospirosis in Wild Animals in Eastern Canada With Particular Attention to the Disease in Rats. *Can J Comp Med Vet Sci, 25*(1), 15-18.

82. Michna, S. W., & Ellis, W. (1974). The isolation of Leptospira belonging to the serogroup ballum from the kidneys of a brown rat (Rattus norvegicus). *Res Vet Sci, 16*(2), 263-264.

83. Middleton, A. D. (1929). Leptospira icterohaemorrhagiae in Oxford Rats. *J Hyg (Lond), 29*(2), 219-226.

84. Millán, J., Cevidanes, A., Chirife, A. D., Candela, M. G., & Leon-Vizcaino, L. (2018). Risk factors of Leptospira infection in Mediterranean periurban micromammals. *Zoonoses Public Health, 65*(1), e79-e85. doi:10.1111/zph.12411

85. Milner, A. R., Wilks, C. R., Spratt, D. M., & Presidente, P. J. (1981). The prevalence of anti-leptospiral agglutinins in sera of wildlife in southeastern Australia. *J Wildl Dis, 17*(2), 197-202.

86. Mohamed-Hassan, S. N., Bahaman, A. R., Mutalib, A. R., & Khairani-Bejo, S. (2010). Serological prevalence of leptospiral infection in wild rats at the National Service Training Centres in Kelantan and Terengganu. *Trop Biomed, 27*(1), 30-32.

87. Mohamed-Hassan, S. N., Bahaman, A. R., Mutalib, A. R., & Khairani-Bejo, S. (2012). Prevalence of Leptospires in rats from selected locations in Peninsular Malaysia. *Research Journal of Animal Sciences, 6*(1), 12-25. doi:10.3923/rjnasci.2012.12.25

88. Montes, A. S., Dimas, J. S., & Preciado Rodriguez, F. J. (2002). Rats and dogs: important vectors of leptospirosis in agricultural areas in Cuidad Guzman, Jalisco. *Rev Cubana Med Trop, 54*(1), 21-23.

89. Morales, G. A., Guzman, V. H., & Beltran, L. E. (1978). Leptospirosis in Colombia: isolation of Leptospira spp. from the kidneys of brown rats (Rattus norvegicus) trapped on infected piggeries. *Trop Anim Health Prod, 10*(2), 121-123. doi:10.1007/BF02235323

90. Munoz-Zanzi, C., Mason, M., Encina, C., Gonzalez, M., & Berg, S. (2014). Household characteristics associated with rodent presence and Leptospira infection in rural and urban communities from Southern Chile. *Am J Trop Med Hyg, 90*(3), 497-506. doi:10.4269/ajtmh.13-0334

91. Natarajaseenivasan, K., Boopalan, M., Selvanayaki, K., Suresh, S. R., & Ratnam, S. (2002). Leptospirosis among rice mill workers of Salem, South India. *Jpn J Infect Dis, 55*(5), 170-173.

92. Natarajaseenivasan, K., Vedhagiri, K., Sivabalan, V., Prabagaran, S. G., Sukumar, S., Artiushin, S. C., & Timoney, J. F. (2011). Seroprevalence of Leptospira borgpetersenii serovar javanica infection among dairy cattle, rats and humans in the Cauvery river valley of southern India. *Southeast Asian J Trop Med Public Health, 42*(3), 679-686.

93. O'Guinn, M. L., Klein, T. A., Lee, J. S., Richards, A. L., Kim, H. C., Ha, S. J., . . . Song, J. W. (2010). Serological surveillance of scrub typhus, murine typhus, and leptospirosis in small mammals captured at firing points 10 and 60, Gyeonggi province, Republic of Korea, 2001-2005. *Vector Borne Zoonotic Dis, 10*(2), 125-133. doi:10.1089/vbz.2008.0123

94. Pagès, F., Larrieu, S., Simoes, J., Lenabat, P., Kurtkowiak, B., Guernier, V., . . . Filleul, L. (2016). Investigation of a leptospirosis outbreak in triathlon participants, Reunion Island, 2013. *Epidemiol Infect, 144*(3), 661-669. doi:10.1017/s0950268815001740

95. Paixão, M. S., Alves-Martin, M. F., Tenorio Mda, S., Starke-Buzetti, W. A., Alves, M. L., da Silva, D. T., . . . Lucheis, S. B. (2014). Serology, isolation, and molecular detection of Leptospira spp. from the tissues and blood of rats captured in a wild animal preservation centre in Brazil. *Prev Vet Med, 115*(1-2), 69-73. doi:10.1016/j.prevetmed.2014.03.016

96. Panti-May, J. A., RRC, D. E. A., Gurubel-Gonzalez, Y., Palomo-Arjona, E., Soda-Tamayo, L., Meza-Sulu, J., . . . Costa, F. (2017). A survey of zoonotic pathogens carried by house mouse and black rat populations in Yucatan, Mexico. *Epidemiol Infect, 145*(11), 2287-2295. doi:10.1017/S0950268817001352

97. Parveen, S. M., Suganyaa, B., Sathya, M. S., Margreat, A. A., Sivasankari, K., Shanmughapriya, S., . . . Natarajaseenivasan, K. (2016). Leptospirosis Seroprevalence Among Blue Metal Mine Workers of Tamil Nadu, India. *Am J Trop Med Hyg, 95*(1), 38-42. doi:10.4269/ajtmh.16-0095

98. Patil, D., Dahake, R., Roy, S., Mukherjee, S., Chowdhary, A., & Deshmukh, R. (2014). Prevalence of leptospirosis among dogs and rodents and their possible role in human leptospirosis from Mumbai, India. *Indian J Med Microbiol, 32*(1), 64-67. doi:10.4103/0255-0857.124319

99. Pellizzaro, M., Conrado, F. O., Martins, C. M., Joaquim, S. F., Ferreira, F., Langoni, H., & Biondo, A. W. (2017). Serosurvey of Leptospira spp. and Toxoplasma gondii in rats captured from two zoos in Southern Brazil. *Rev Soc Bras Med Trop, 50*(6), 857-860. doi:10.1590/0037-8682-0138-2017

100. Perez, J., Brescia, F., Becam, J., Mauron, C., & Goarant, C. (2011). Rodent abundance dynamics and leptospirosis carriage in an area of hyper-endemicity in New Caledonia. *PLoS Negl Trop Dis, 5*(10), e1361. doi:10.1371/journal.pntd.0001361

101. Pezzella, M., Lillini, E., Sturchio, E., Ierardi, L. A., Grassi, M., Traditi, F., & Cristaldi, M. (2004). Leptospirosis survey in wild rodents living in urban areas of Rome. *Ann Ig, 16*(6), 721-726.

102. Priya, C. G., Hoogendijk, K. T., Berg, M., Rathinam, S. R., Ahmed, A., Muthukkaruppan, V. R., & Hartskeerl, R. A. (2007). Field rats form a major infection source of leptospirosis in and around Madurai, India. *J Postgrad Med, 53*(4), 236-240. doi:10.4103/0022-3859.37511

103. Pui, C. F., Bilung, L. M., Apun, K., & Su'ut, L. (2017). Diversity of Leptospira spp. in Rats and Environment from Urban Areas of Sarawak, Malaysia. *J Trop Med, 2017*, 3760674. doi:10.1155/2017/3760674

104. Rahelinirina, S., Leon, A., Harstskeerl, R. A., Sertour, N., Ahmed, A., Raharimanana, C., . . . Cornet, M. (2010). First isolation and direct evidence for the existence of large small-mammal reservoirs of Leptospira sp. in Madagascar. *PLoS One, 5*(11), e14111. doi:10.1371/journal.pone.0014111

105. Ralaiarijaona, R. L., Bellenger, E., Chanteau, S., Roger, F., Perolat, P., & Rasolofo Razanamparany, V. (2001). Detection of leptospirosis reservoirs in Madagascar using the polymerase chain reaction technique. *Arch Inst Pasteur Madagascar, 67*(1-2), 34-36.

106. Rim, B. M., Rim, C. W., Chang, W. H., & Kakoma, I. (1993). Leptospirosis serology in Korean wild animals. *J Wildl Dis, 29*(4), 602-603. doi:10.7589/0090-3558-29.4.602

107. Rislakki, V., & Salminen, A. (1955). Investigations of leptospirosis in rats in Finland. *Acta Pathol Microbiol Scand, 37*(1), 121-131. doi:10.1111/j.1699-0463.1955.tb00927.x

108. Romero-Vivas, C. M., Cuello-Perez, M., Agudelo-Florez, P., Thiry, D., Levett, P. N., & Falconar, A. K. (2013). Cross-sectional study of Leptospira seroprevalence in humans, rats, mice, and dogs in a main tropical sea-port city. *Am J Trop Med Hyg, 88*(1), 178-183. doi:10.4269/ajtmh.2012.12-0232

109. Rust, J. H. (1948). Leptospirosis in Puerto Rican wild rats. *PR J Public Health Trop Med, 24*(2), 105-112 Also Spanish transl , 113-120.

110. Saito, M., Villanueva, S. Y., Masuzawa, T., Haraguchi, Y., Ita, S., Miyahara, S., . . . Yoshida, S. (2015). The usefulness of semi-solid medium in the isolation of highly virulent Leptospira strains from wild rats in an urban area of Fukuoka, Japan. *Microbiol Immunol, 59*(6), 322-330. doi:10.1111/1348-0421.12260

111. Sames, W. J., Klein, T. A., Kim, H. C., Gu, S. H., Kang, H. J., Shim, S. H., . . . Song, J. W. (2010). Serological surveillance of scrub typhus, murine typhus, and leptospirosis in small mammals captured at Twin Bridges Training Area, Gyeonggi Province, Republic of Korea, 2005-2007. *Mil Med, 175*(1), 48-54. doi:10.7205/MILMED-D-05-1308

112. Samir, A., Soliman, R., El-Hariri, M., Abdel-Moein, K., & Hatem, M. E. (2015). Leptospirosis in animals and human contacts in Egypt: broad range surveillance. *Rev Soc Bras Med Trop, 48*(3), 272-277. doi:10.1590/0037-8682-0102-2015

113. Santos, A. A., Figueira, C. P., dos Reis, M. G., Costa, F., & Ristow, P. (2015). Heterogenic colonization patterns by Leptospira interrogans in Rattus norvegicus from urban slums. *Braz J Microbiol, 46*(4), 1161-1164. doi:10.1590/S1517-838246420140873

114. Scialfa, E., Bolpe, J., Bardon, J. C., Ridao, G., Gentile, J., & Gallicchio, O. (2010). Isolation of Leptospira interrogans from suburban rats in Tandil, Buenos Aires, Argentina. *Rev Argent Microbiol, 42*(2), 126-128. doi:10.1590/S0325-75412010000200012

115. Sharma, S., Vijayachari, P., Sugunan, A. P., & Sehgal, S. C. (2003). Leptospiral carrier state and seroprevalence among animal population--a cross-sectional sample survey in Andaman and Nicobar Islands. *Epidemiol Infect, 131*(2), 985-989.

116. Shimizu, M. M. (1984). Environmental and biological determinants for the prevalence of leptospirosis among wild small mammal hosts, island of Hawaii. *Int J Zoonoses, 11*(2), 173-188.

117. Smith, C. E., Turner, L. H., Harrison, J. L., & Broom, J. C. (1961). Animal leptospirosis in Malaya: 3. Incidence in rats by sex, weight and age. *Bull World Health Organ, 24*(6), 807-816.

118. Socolovschi, C., Angelakis, E., Renvoise, A., Fournier, P. E., Marie, J. L., Davoust, B., . . . Raoult, D. (2011). Strikes, flooding, rats, and leptospirosis in Marseille, France. *Int J Infect Dis, 15*(10), e710-715. doi:10.1016/j.ijid.2011.05.017

119. Strand, T. M., Lohmus, M., Persson Vinnersten, T., Rasback, T., Sundstrom, K., Bergstrom, T., & Lundkvist, A. (2015). Highly Pathogenic Leptospira Found in Urban Brown Rats (Rattus norvegicus) in the Largest Cities of Sweden. *Vector Borne Zoonotic Dis, 15*(12), 779-781. doi:10.1089/vbz.2015.1800

120. Suepaul, S. M., Carrington, C. V., Campbell, M., Borde, G., & Adesiyun, A. A. (2010). Serovars of Leptospira isolated from dogs and rodents. *Epidemiol Infect, 138*(7), 1059-1070. doi:10.1017/s0950268809990902

121. Suepaul, S. M., Carrington, C. V., Campbell, M., Borde, G., & Adesiyun, A. A. (2014). Seroepidemiology of leptospirosis in dogs and rats in Trinidad. *Trop Biomed, 31*(4), 853-861.

122. Sulzer, C. R., Harvey, T. W., & Galton, M. M. (1968). Comparison of diagnostic technics for the detection of leptospirosis in rats. *Health Lab Sci, 5*(3), 171-173.

123. Sumanta, H., Wibawa, T., Hadisusanto, S., Nuryati, A., & Kusnanto, H. (2015). Genetic variation of Leptospira isolated from rats catched in Yogyakarta Indonesia. *Asian Pac J Trop Med, 8*(9), 710-713. doi:10.1016/j.apjtm.2015.07.029

124. Sunbul, M., Esen, S., Leblebicioglu, H., Hokelek, M., Pekbay, A., & Eroglu, C. (2001). Rattus norvegicus acting as reservoir of leptospira interrogans in the Middle Black Sea region of Turkey, as evidenced by PCR and presence of serum antibodies to Leptospira strain. *Scand J Infect Dis, 33*(12), 896-898. doi:10.1080/00365540110076796

125. Taylor, K. D., Turner, L. H., & Everard, J. D. (1991). Leptospires in Rattus spp. on Barbados. *J Trop Med Hyg, 94*(2), 102-103.

126. Theuerkauf, J., Perez, J., Taugamoa, A., Niutoua, I., Labrousse, D., Gula, R., . . . Goarant, C. (2013). Leptospirosis risk increases with changes in species composition of rat populations. *Naturwissenschaften, 100*(4), 385-388. doi:10.1007/s00114-013-1033-6

127. Torres-Castro, M., Guillermo-Cordero, L., Hernandez-Betancourt, S., Gutierrez-Ruiz, E., Agudelo-Florez, P., Pelaez-Sanchez, R., . . . Puerto, F. I. (2016). First histopathological study in kidneys of rodents naturally infected with Leptospira pathogenic species from Yucatan, Mexico. *Asian Pac J Trop Med, 9*(2), 145-147. doi:10.1016/j.apjtm.2016.01.018

128. Tucunduva de Faria, M., Athanazio, D. A., Goncalves Ramos, E. A., Silva, E. F., Reis, M. G., & Ko, A. I. (2007). Morphological alterations in the kidney of rats with natural and experimental Leptospira infection. *J Comp Pathol, 137*(4), 231-238. doi:10.1016/j.jcpa.2007.08.001

129. Vado-Solis, I., Cardenas-Marrufo, M. F., Jimenez-Delgadillo, B., Alzina-Lopez, A., Laviada-Molina, H., Suarez-Solis, V., & Zavala-Velazquez, J. E. (2002). Clinical-epidemiological study of leptospirosis in humans and reservoirs in Yucatan, Mexico. *Rev Inst Med Trop Sao Paulo, 44*(6), 335-340.

130. Van der Hoeden, J., & Szenberg, E. (1964). Leptospira Infections in Rats in Israel. *Trop Geogr Med, 16*, 377-384.

131. Vanasco, N. B., Rossetti, C., Sequeira, G., Sequeira, M. D., Calderon, G., & Tarabla, H. D. (2000). First isolations of leptospires serogroup Ballum serovar arborea in Argentina. *Vet Rec, 147*(9), 246-247. doi:10.1136/vr.147.9.246

132. Vanasco, N. B., Sequeira, M. D., Sequeira, G., & Tarabla, H. D. (2003). Associations between leptospiral infection and seropositivity in rodents and environmental characteristics in Argentina. *Prev Vet Med, 60*(3), 227-235. doi:https://doi.org/10.1016/S0167-5877(03)00144-2

133. Vedhagiri, K., Natarajaseenivasan, K., Prabhakaran, S. G., Selvin, J., Narayanan, R., Shouche, Y. S., . . . Ratnam, S. (2010). Characterization of leptospira borgpetersenii isolates from field rats (rattus norvegicus) by 16s rrna and lipl32 gene sequencing. *Braz J Microbiol, 41*(1), 150-157. doi:10.1590/S1517-838220100001000022

134. Villanueva, M. A., Mingala, C. N., Gloriani, N. G., Yanagihara, Y., Isoda, N., Nakajima, C., . . . Koizumi, N. (2016). Serological investigation of Leptospira infection and its circulation in one intensive-type water buffalo farm in the Philippines. *Jpn J Vet Res, 64*(1), 15-24. doi:10.14943/jjvr.64.1.15

135. Villanueva, S. Y., Ezoe, H., Baterna, R. A., Yanagihara, Y., Muto, M., Koizumi, N., . . . Yoshida, S. (2010). Serologic and molecular studies of Leptospira and leptospirosis among rats in the Philippines. *Am J Trop Med Hyg, 82*(5), 889-898. doi:10.4269/ajtmh.2010.09-0711

136. Vimala, G., Rani, A. M., & Gopal, V. R. (2014). Leptospirosis in vellore: a clinical and serological study. *Int J Microbiol, 2014*, 643940. doi:10.1155/2014/643940

137. Vitale, M., Di Bella, C., Agnello, S., Curro, V., Vicari, D., & Vitale, F. (2007). Leptospira interrogans survey by PCR in wild rodents coming from different urban areas of Palermo, Italy. *Rev Cubana Med Trop, 59*(1), 59-60.

138. Vitale, M., Agnello, S., Chetta, M., Amato, B., Vitale, G., Bella, C. D., . . . Presti, V. (2018). Human leptospirosis cases in Palermo Italy. The role of rodents and climate. *J Infect Public Health, 11*(2), 209-214. doi:10.1016/j.jiph.2017.07.024

139. Wangroongsarb, P., Saengsongkong, W., Petkanjanapong, W., Mimgratok, M., Panjai, D., Wootta, W., & Hagiwara, T. (2008). An application of duplex PCR for detection of Leptospira spp. and Orientia tsutsugamushi from wild rodents. *Jpn J Infect Dis, 61*(5), 407-409.

140. Webster, J. P., Ellis, W. A., & Macdonald, D. W. (1995). Prevalence of Leptospira spp. in wild brown rats (Rattus norvegicus) on UK farms. *Epidemiol Infect, 114*(1), 195-201.

141. Wong, M., Katz, A. R., Li, D., & Wilcox, B. A. (2012). Leptospira infection prevalence in small mammal host populations on three Hawaiian islands. *Am J Trop Med Hyg, 87*(2), 337-341. doi:10.4269/ajtmh.2012.12-0187

142. Wongbutdee, J., & Jittimanee, J. (2016). Detection of Leptospira in Rats Trapped from Households in Phraroj Village, Muang Sam Sip District, Ubon Ratchathani Province Using Polymerase Chain Reaction Technique. *J Med Assoc Thai, 99 Suppl 1*, S17-21.

143. Yalin, W., Lingbing, Z., Hongliang, Y., Jianmin, X., Xiangyan, Z., Xiaokui, G., . . . Jinhong, Q. (2011). High prevalence of pathogenic Leptospira in wild and domesticated animals in an endemic area of China. *Asian Pac J Trop Med, 4*(11), 841-845. doi:10.1016/S1995-7645(11)60205-8

144. Zhou, J., Huang, X., He, H., Zhang, X., Liu, A., Yang, T., . . . Tan, H. (2009). Epidemiological study on leptospirosa infection of host animals and healthy population in flood areas. *Zhong Nan Da Xue Xue Bao Yi Xue Ban, 34*(2), 99-103.

145. Zilber, A. L., Belli, P., Artois, M., Kodjo, A., & Djelouadji, Z. (2016). First Observation of Leptospira interrogans in the Lungs of Rattus norvegicus. *Biomed Res Int, 2016*, 9656274. doi:10.1155/2016/9656274
