# Supplementary material for: Leptospira infection in rats: A literature review of global prevalence and distribution
Source: PLoS Negl Trop Dis. 2019 Aug 9;13(8):e0007499. doi: 10.1371/journal.pntd.0007499 (PMC6688788; doi:10.1371/journal.pntd.0007499)
Supplement: S1 Map — (HTML) [file pntd.0007499.s007.html]

S1 Map (Lepto Review)
